# Comparison of Quasispecies Diversity of HCV between Chronic Hepatitis C and Hepatocellular Carcinoma by Ultradeep Pyrosequencing

**DOI:** 10.1155/2014/853076

**Published:** 2014-06-05

**Authors:** Chang-Wook Park, Min-Chul Cho, Keumrock Hwang, Sun-Young Ko, Heung-Bum Oh, Han Chu Lee

**Affiliations:** ^1^Department of Laboratory Medicine, Asan Medical Center, University of Ulsan College of Medicine, 88 Olympic-ro 43-gil, Songpa-gu, Seoul 138-736, Republic of Korea; ^2^Department of Laboratory Medicine, Gyeongsang National University Hospital and Gyeongsang National University School of Medicine, Jinju 660-701, Republic of Korea; ^3^Department of Laboratory Medicine, Sure Quest Laboratory, Yongin 446-916, Republic of Korea; ^4^Department of Laboratory Medicine, College of Medicine, Korea University, Seoul 136-705, Republic of Korea; ^5^Department of Internal Medicine, Asan Medical Center, University of Ulsan College of Medicine, Seoul 138-736, Republic of Korea

## Abstract

*Backgrounds.* Hepatitis C virus (HCV) exists as population of closely related genetic variants known as quasispecies. HCV quasispecies diversity is strongly influenced by host immune pressure on virus. Quasispecies diversity is expected to decline as host immune response to HCV decreases over natural course of progressing from chronic hepatitis C (CHC) to hepatocellular carcinoma (HCC). *Methods.* Ultradeep pyrosequencing (UDPS) was used to evaluate degree of quasispecies diversity in 49 patients infected with HCV including 26 with CHC and 23 with HCC. Whole structural protein of HCV genome was subjected to UDPS. *Results.* Shannon's indices for quasispecies diversity in HCV E1 were significantly lower in patients with HCC than in those with CHC. 14 amino acid positions differed significantly between two groups. Area under curve of ROC analysis for differentiating HCC from CHC was >0.8 for all of 14 amino acid positions. *Conclusion.* HCV quasispecies diversity as indicator of declining host immune functions was easily assessed by UDPS technology. Shannon's indices in 14 amino acid positions were found to differentiate between patients with CHC and those with HCC. Our data propose that degree of HCV quasispecies measured by UDPS might be useful to predict progression of HCC in chronic HCV patients.

## 1. Introduction

Hepatitis C virus (HCV) is an enveloped RNA virus [1]. HCV is capable of producing highly diverse variants, so called quasispecies, by initiating replications based on RNA-dependent, error-prone RNA polymerase [2, 3]. These HCV quasispecies are considered as one of the mechanisms by which HCV evades host immune pressure [4].

Nearly 80% of the patients with HCV infection progress to chronic HCV infection. Of those, 20% develop cirrhosis over a period of 20 years. The risk of developing hepatocellular carcinoma (HCC) increases by 5% per year in those with cirrhosis [5–7]. It is known that the progression of chronic hepatitis C to HCC is accompanied by anergy of host immune system [8]. These changes in host immune response to the virus also affect the diversity in HCV quasispecies.

A lower HCV quasispecies diversity has been expected in patients with HCC than those with chronic HCV infection. However, the results of earlier studies have not been consistent with this expectation [9, 10]. Divided results are attributed to the fact that the numbers of samples and clones analyzed in cloning and sequencing analysis were not enough [9, 10] and that single stranded conformation polymorphism (SSCP) and heteroduplex tracking assays (HTA) used in those studies had technical shortfalls [11–15]. Also, investigated HCV positions varied among studies [9, 10].

The next generation sequencing (NGS) that allows massive parallel sequencing has been recently developed. Massive parallel sequencing allows a simultaneous sequencing by breaking the whole genome into numerous pieces, generating the results of hundreds of megabase sequences in a single sequencing run [16–18]. Using these technologies, ultradeep pyrosequencing (UDPS) has been used to detect various genetic variants by analyzing sequences from the same gene regions. This tool is very effective to detect variants of viral quasispecies [19–22]. UDPS of HCV quasispecies offers the same benefits as the analysis of several thousands of clones at a time, providing more accurate and sensitive results on quasispecies diversity. In this study, thus, UDPS was performed to investigate the degree of quasispecies diversity between the patients with chronic HCV infection and those with HCC in the whole HCV structural proteins.

## 2. Materials and Methods

### 2.1. Study Subjects

A total of 49 samples with HCV genotype 1b were included for this study. Among them, 26 patients had chronic HCV infection and 23 had HCV-induced hepatocellular carcinoma. *t*-tests were performed to evaluate differences between the two groups in demographic profile (age and gender), the quantity of HCV RNA, and biochemical profile (AST, ALT, albumin, platelet, bilirubin, and prothrombin time).

The quantity of HCV RNA was measured using a real-time PCR assay (Abbott Molecular Inc., Abbott Park, IL, USA) following the manufacturer's recommendations. HCV RNA level of all samples was over 1.0 × 10^4^ IU/mL.

The study protocol was reviewed and approved by Asan Medical Center Institutional Review Board, Ethics reference number (2013-0643). According to the policy in our institutional review board, informed consent was waived since the remaining serum samples with which HCV genotyping tests were already completed and reported were utilized in this study, and all the patient information was kept anonymous and minimally used only for the study purpose. The recommendations of the Declaration of Helsinki for biomedical research involving human subjects were followed.

### 2.2. PCR and UDPS

HCV RNA was isolated using QIAamp MinElute Virus Spin Kit (Qiagen Inc., Valencia, CA, USA) according to the manufacturer's recommendations. Obtained HCV RNA was synthesized to cDNA and then amplified twice to cover a 2 kb fragment of core and E1 and E2 regions using four primer pairs (Table 1). The primary PCR was performed with 5 *μ*L of HCV cDNA and 20 *μ*L of polymerase chain reaction reagent. Advantage 2 PCR kit (Clontech, Inc., CA, USA) was used as polymerase chain reaction solution, which contains 1x PCR buffer 40 mM Tricine-KOH (pH 8.7), 15 mM KOAc, 3.5 mM Mg(OAc)2, 3.75 *μ*g/mL BSA, Tween 20 0.005%, 25 mM dNTP, 12.5 *μ*M primer, and 1x Advantage 2 polymerase mix. PCR reactions started with predenaturation step at 95°C for 10 minutes, followed by denaturation at 95° for 30 seconds, annealing at 55.8°C for one minute, 25 cycles of extension at 72°C for one minute, and additional extension at 72°C for 10 minutes. The secondary PCR was performed with 5 *μ*L of primary PCR and 45 *μ*L of polymerase chain reaction solution. Composition of polymerase chain reaction reagent was the same as that of primary PCR. The secondary reactions also included predenaturation step at 95°C for 10 minutes, denaturation at 95° for 30 seconds, annealing at 55.8°C for one minute, 25 cycles of extension at 72°C for one minute, and additional extension at 72°C for 10 minutes.

PCR amplification yield was purified with AMPure beads (Beckman Coulter Inc., Brea, CA, USA). And UDPS was performed in a sequential order of library preparation based on GS FLX Titanium platform, emulsion PCR, and pyrosequencing. DNA library was quantified using RiboGreen (Invitrogen, Eugene, OR, USA). Each library was diluted to 2 × 10^6^ molecules/*μ*L, and the entire volume of 20 *μ*L from each sample was used to make pooled library. Emulsion PCR was performed and particles were counted using Multisizer 3 Coulter Counter (Beckman Coulter Inc.). After this, pyrosequencing was performed on region 1/4 of a 70 mm × 75 mm Picotiter Plate (PTP) using GS FLX 454 Genome Sequencer according to the manufacturer's recommendations.

### 2.3. Analysis of Sequence Data Generated by UDPS

After UDPS, the standard strain of HCV was set to align each UDPS read to a sequence. The standard sequence of HCV was defined by aligning 388 whole genome sequences of HCV genotype 1b, which were obtained from Los Alamos National Laboratory (http://hcv.lanl.gov). The standard sequence was applied when 70% or higher identical nucleotides were observed in a specific sequence position. If not, the standard sequence was marked with “N.” If 70% or higher nucleotides were blank in a specific sequence position, the corresponding position was eliminated from the standard sequence. The standard amino acid sequence was deduced by replacing the sequence of protein production with the amino acids sequence in the standard sequence of HCV.

UDPS reads were aligned along with the standard sequence using Amplicon Variant Analyzer (AVA), supplied by the manufacturer. A sequence analysis program was developed to determine HCV quasispecies diversity. This analysis program is designed to store aligning reads in a database and evaluate the diversity of HCV quasispecies after calculating Shannon diversity index for the nucleotides and amino acids sequences, respectively.

The equation to calculate Shannon diversity index (*H*) for the nucleotides and amino acids sequences is as follows:

(1) H=−∑iSpiln⁡⁡pi

In this equation, the summation from *i* to *S* is from 1 to 4 for nucleotide and from 1 to 20 for amino acid considering the number of nucleotide and amino acid, respectively. Thus, *P*_*i*_ represents proportion of each nucleotide and amino acid at each position.

**Figure 1:**
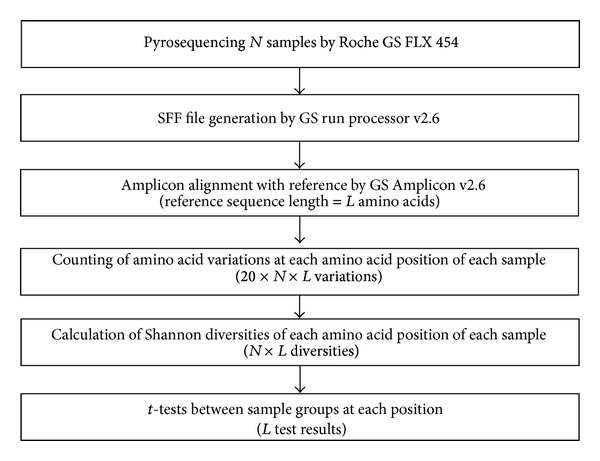
The workflow of UDPS data treatment and statistical process in this study.

*t*-test was also done for the nucleotides and amino acid positions to evaluate the differences in the HCV quasispecies diversity between the chronic HCV infection group and the hepatocellular carcinoma group. Bonferroni correction was used to verify statistical significance of the results of *t*-tests and corrected *P* value of less than 0.05 is considered significant. Receiver operating characteristic (ROC) curves were made for each nucleotide and amino acid sequence position showing significant differences between the two groups. Specificity and area under the curve (AUC) were calculated for the positions showing sensitivity of 90% or higher in the ROC curve. All the workflow including UDPS data treatment and statistical process is summarized in Figure 1.

## 3. Results

### 3.1. Study Subjects

A total of 26 patients in the chronic HCV infection group showed mean ± SD of 58 ± 13, 1.9 ± 1.6, 76.0 ± 71.1, 79.0 ± 71.1, 4.0 ± 0.4, 158 ± 75, 1.1 ± 0.4, and 97.5 ± 17.3, respectively, in age, the quantity of HCV RNA (×10^6^ IU/mL), AST, ALT, albumin, platelet count (×10^3^), bilirubin, and prothrombin time (%). A total of 26 patients in the HCC group exhibited 65 ± 9, 1.5 ± 2.7, 79.4 ± 40.0, 72.0 ± 58.0, 3.2 ± 0.5, 92 ± 38, 1.5 ± 0.7, and 74.4 ± 18.3 in the same categories. Significant differences were observed in age, albumin, platelet, bilirubin, and prothrombin time between the two groups (*P* < 0.05). But no significant differences were found in gender, HCV RNA quantity, AST, and ALT.

### 3.2. Results of UDPS

**Figure 2:**
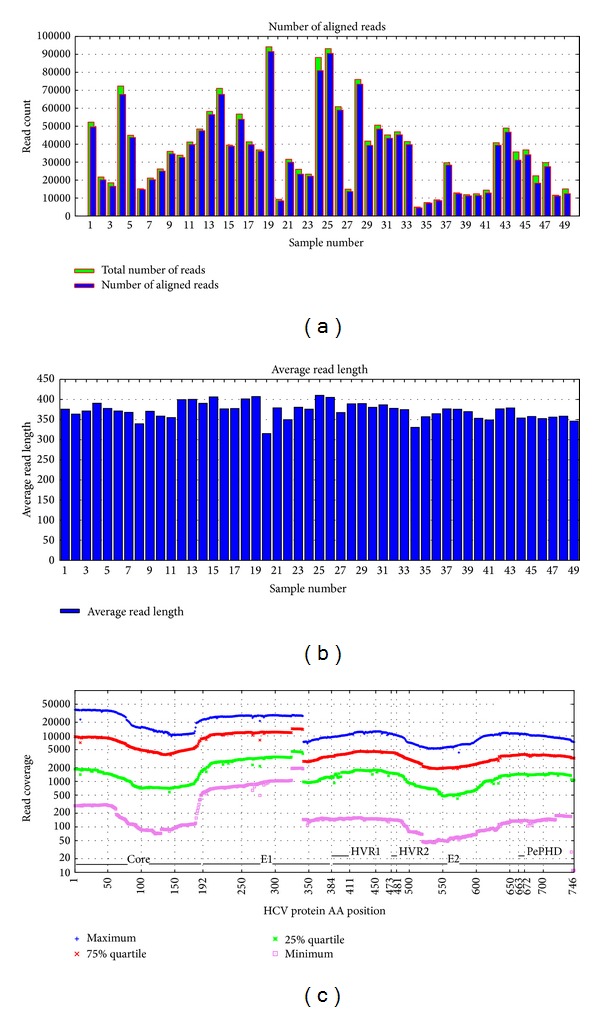
A summary of ultradeep pyrosequencing (UDPS) results. (a) Number of reads and aligned reads of each sample. (b) Average read length of each sample. (c) Hepatitis C virus (HCV) genome coverage by ultradeep pyrosequencing. The dots represent minimum, 25% quartile, 75% quartile, and maximum coverage of reads of samples at each amino acid position of HCV protein. HVR: hypervariable region.

A total of 1,822,601 sequence reads were obtained from 49 samples using UDPS in the average number of sequence reads as 37,196 (range 4,996–94,180) per sample. Of those, UDPS sequence reads that can be aligned with standard sequence were 35,311 in average per sample, accounting for 94% of total reads (Figure 2(a)), and the average read length was 350 bp per each sample (Figure 2(b)). The number of reads at each position in HCV genome (HCV genome coverage) was nearly 2,000 to 10,000 (Figure 2(c)).

### 3.3. HCV Quasispecies Diversity of the Patients with CHC and Those with HCC Caused by HCV Infection

**Figure 3:**
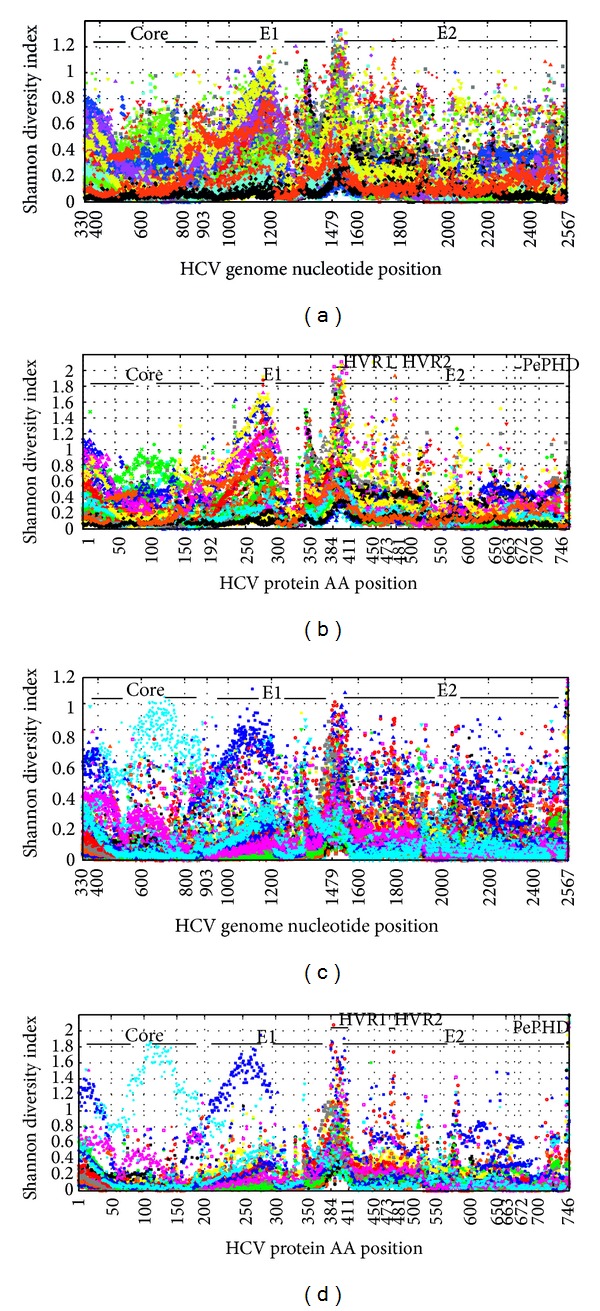
Hepatitis C virus (HCV) quasispecies diversity in the patients with chronic hepatitis C (CHC) and those with hepatocellular carcinoma (HCC) caused by HCV infection. (a) HCV quasispecies diversity of each nucleotide position in patients with CHC; (b) HCV quasispecies diversity of each amino acid position in patients with CHC; (c) HCV quasispecies diversity of each nucleotide position in patients with HCC caused by HCV infection; (d) HCV quasispecies diversity of each amino acid position in patients with HCC caused by HCV infection. HVR: hypervariable region; AA: amino acid.

The degree of HCV quasispecies diversity was analyzed for the whole structural protein of the HCV genome by calculating Shannon's diversity index for each of the nucleotides and amino acids sequence positions (Figure 3).

**Figure 4:**
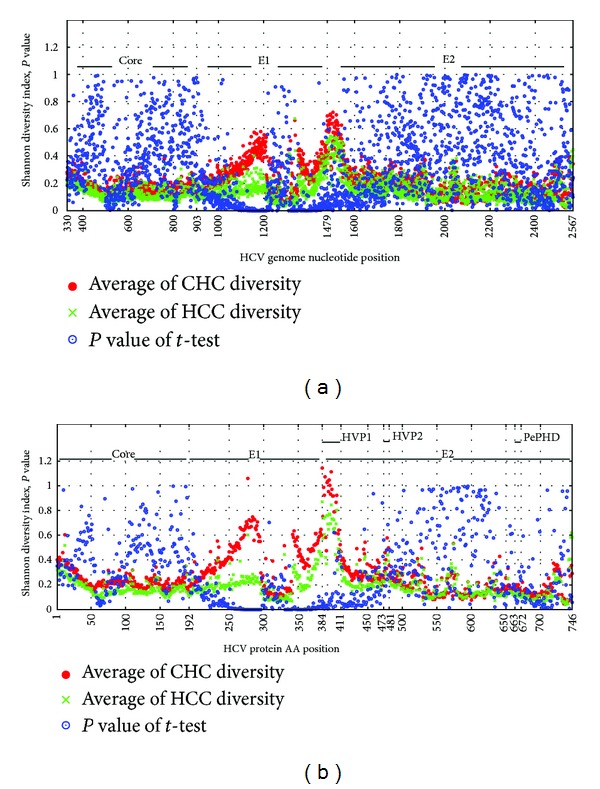
Comparison of HCV quasispecies diversity in chronic hepatitis C (CHC) and hepatocellular carcinoma (HCC) caused by HCV. Red dots represent the average Shannon diversity indices of the patients with CHC. Green dots represent the average Shannon diversity indices of the patients with HCC caused by HCV infection. And blue dots represent the corrected *P* value. (a) Comparison of HCV quasispecies diversity in each nucleotide position of HCV structural protein. (b) Comparison of HCV quasispecies diversity in each amino acid position of HCV structural protein.

HCV quasispecies diversity of the HCV structural protein was significantly lower in the HCC group compared to the chronic HCV infection group. Especially, HCV quasispecies diversity has more significantly decreased at E1 position in the HCC group than the chronic HCV infection group (Figure 4).

**Figure 5:**
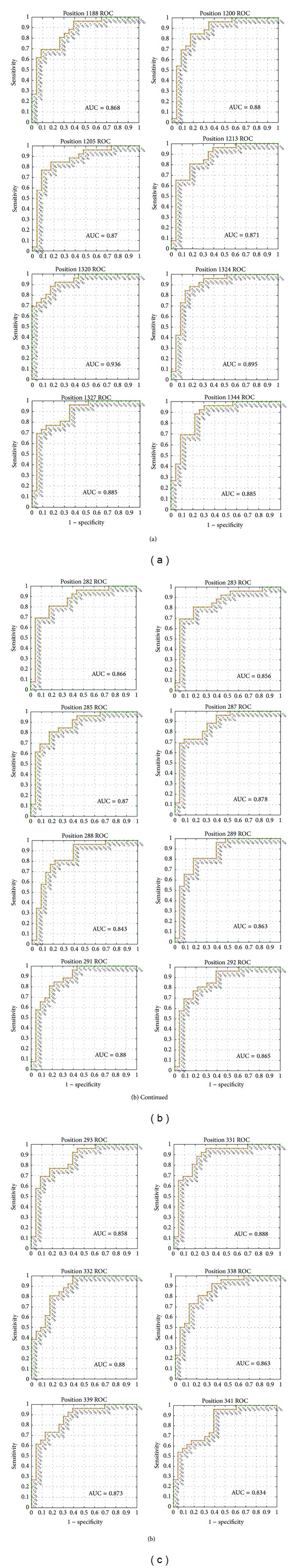
Receiver operating characteristic (ROC) curves of 8 nucleotide positions and 14 amino acid positions. (a) ROC curves of 8 nucleotide positions showing significantly different diversities between the patients with chronic hepatitis C and those with hepatocellular carcinoma caused by HCV infection. (b) ROC curves of 14 amino acid positions showing significantly different diversities between the patients with chronic hepatitis C without liver cirrhosis and those with hepatocellular carcinoma caused by HCV infection. AUC: area under the curve.

A total of eight nucleotide positions showed statistically significant differences between the two groups (Table 2). Analysis of ROC curve for each of the eight significant nucleotide positions revealed that the chronic HCV infection group and the HCC group could be differentiated from each other in AUC value of 0.8 or higher (Figure 5(a)). In addition, a total of 14 amino acid positions exhibited statistically significant differences between the two groups (Table 3). Analysis of ROC curve for each of the 14 significant amino acid positions revealed that two groups could be discriminated against each other in AUC value of 0.8 or higher (Figure 5(b)).

## 4. Discussion

It has been expected that the diversity of HCV quasispecies would be decreased with disease progression due to reduced immune response, while the diversity of HCV quasispecies is increased by active immune responses in early phase. However, previous study results have not been consistent with this expectation when HCV quasispecies diversity was measured by clone-based sequencing, SSCP, or HTA. Even though cloning and sequencing method of the quasispecies variants offers an advantage of identifying their whole characteristics, this approach is rather complicated, demanding, and costly. Therefore, the number of clones that can be obtained is limited. SSCP method also makes it difficult to optimize the experimental condition. Even more, the diversity of viral quasispecies was assessed depending only on a small number of SSCP bands ranging from 4 to 20 [11–14, 23]. HTA method is less expensive than the clone-based sequencing and offers higher sensitivity than SSCP. It is also easy to optimize experimental conditions. However, like SSCP, it does not enable researchers to identify the location of each variant and the proportion of variants [24].

Newly developed UDPS method, on the contrary, is expected to overcome the pitfalls of above mentioned methods by enabling us to analyze simultaneously thousands of clonally amplified gene fragments. This improves the reliability of the results for the proportion of each variant and thus for the diversity measures. In this study, the average UDPS reads exceeded 2,000 for core and E1 and E2 proteins, which is much larger than those clones previously reported in other studies. Honda et al. analyzed 10 clones per sample. Hayashi et al. generated five clones per sample, Curran et al. 373 from 39 samples, and Qin et al. 767 clones from about 100 samples [9, 10, 25, 26]. Of those studies, Qin et al. who analyzed the largest number of clones reported that HCV quasispecies diversity decreased over the natural course of HIV infection [9]. In the probabilistic view, approximately 250 clones should be generated per sample in order to include more than 99% of clones, each of which comprises more than 2% of total clones. In our study, more than 2,000 clones were analyzed, which implies that almost all types of clones present in serum were included in the analysis.

Host's humoral immune system attacks or neutralizes viral envelope proteins, which make the envelope hypervariable in the process of escaping the host immunity. Most of recent studies [9, 10] mainly focused on the analysis of HCV quasispecies diversity on E2 since this region has been known to harbor the most severe variations. However, this study analyzed a wide range of structural proteins, including core and E1 and E2, and found unexpectedly that E1 diversities rather than E2 were lower in the HCC group than that of chronic HCV infection group. An interesting finding was that all amino acid positions showing statistical differences between the two groups matched well with cytotoxic T-cell epitopes, which was claimed by Yusim et al. [27]. The fact that host immune response to HCV decreases irrespective of decreased diversity in T-cell epitope supports the idea that cytotoxic T-cells specific to HCV are more anergic in HCC than in chronic hepatitis C. And our findings could imply that the loss of cytotoxic T lymphocyte response may play an important role in carcinogenesis of HCC by HCV.

HCC can occur in HCV infection only after liver cirrhosis stage, the advanced fibrotic stage. Thus, it is recommended to perform surveillance for the occurrence of HCC since surgical treatment is only available for those with small-sized cancer and early stage. Currently, abdominal ultrasound and* alpha* fetoprotein (AFP) tests are recommended for patients with cirrhosis every six months [28]. However, AFP is known to be less sensitive and to have low positive predictive value especially in viral hepatocellular carcinoma. The diagnostic sensitivity of AFP is estimated at only 25% in those with tumor size of 3 cm or smaller, the resectable size, and nearly 50% in those with 3 cm or larger and specificity range from 76% to 96% [29, 30]. Therefore, new serum markers are actively being investigated and introduced for HCC detection [31]. Des-r-carboxyprothrombin (DCP) called PIVKA-II is one of the widely used additional markers for hepatocellular carcinoma. However, serum DCP level increases only in 50% to 60% of patients with hepatocellular carcinoma. The change in DCP value is less significant in patients with early hepatocellular carcinomas because only 15% to 30% of patients showed elevated DCP value [32]. DCP sensitivity ranged from 48% to 62%, and DCP specificity was reported in a range of 81 to 92%. Thus, it is known that DCP's diagnostic value as a hepatocellular carcinoma marker is similar to that of AFP [33]. In addition, a-l-fucosidase, r-glutamyl transferase, glypican-3, and squamous cell carcinoma antigen are used as a marker. However, no specific marker is satisfactory in terms of sensitivity and specificity. The next generation sequencing (NGS) is being increasingly used in clinical laboratories. Thus, Shannon's diversity index for 14 amino acid positions which show >0.8 area under curve of the ROC curve could be feasible as one of the new biomarkers in predicting the progression of hepatocellular carcinoma in chronic HCV patients.

In conclusion, this study found a significant decrease of HCV quasispecies diversity in the E1 region in patients with HCV-induced HCC. 14 amino acid positions were identified where patients with HCC could be differentiated from those with chronic HCV infection. These amino acid positions were matched with cytotoxic T-cell epitopes, which reinforces earlier findings that HCV-specific T-cells become anergic over the natural courses of chronic HCV infection. Our data also propose that the degree of HCV quasispecies diversity measured by UDPS might provide useful information to predict the progression of hepatocellular carcinoma in chronic HCV patients.

## Figures and Tables

**Table 1:** Primer sequences for PCR amplification of HCV genome encoding structural proteins.

Region	Primer name		Primer sequence (5′ → 3′)
Core	1st	B1xL.1-AP1	GGGCGACACTCCACCATAG
		B1xR.1-AP1	GCCGGCRAARAGYAGCAYC
	2nd	B1xL.1-AP2	ACTCCCCTGTGAGGAACTAY
		B1xR.1-AP3	TGGGATCCGGAGYARCTG

E1/E2	1st	B1yL.1-AP1	TTCTGYTCCGCYATGTAYG
		B1yR.1-AP1	CACAAGRAAGGAGAGRANRC
	2nd	B1yL.1-AP3	GAACTGGTCRCCYACARCRG
		B1yR.1-AP2	GGACCACCARGTTCTCYARG

**Table 2:** Eight nucleotide positions showing significantly different diversity between the patients with chronic hepatitis C (CHC) and those with hepatocellular carcinoma (HCC) caused by HCV infection.

Nucleotide position	Shannon diversity index		*P* value	AUC by ROC
	CHC (mean ± SD)	HCC (mean ± SD)		
1188	0.494 ± 0.250	0.174 ± 0.178	0.014	0.868
1200	0.504 ± 0.245	0.176 ± 0.200	0.014	0.88
1205	0.477 ± 0.212	0.186 ± 0.183	0.012	0.87
1213	0.392 ± 0.188	0.135 ± 0.167	0.017	0.871
1320	0.131 ± 0.058	0.048 ± 0.024	<0.001	0.936
1324	0.117 ± 0.041	0.056 ± 0.035	0.003	0.895
1327	0.121 ± 0.049	0.060 ± 0.035	0.009	0.885
1344	0.098 ± 0.042	0.044 ± 0.028	0.008	0.885

**Table 3:** Fourteen amino acid positions showing significantly different diversity between the patients with chronic hepatitis C (CHC) and those with hepatocellular carcinoma (HCC) caused by HCV infection.

Amino acid position	Shannon diversity index		*P* value	AUC by ROC
	CHC (mean ± SD)	HCC (mean ± SD)		
282	0.669 ± 0.403	0.220 ± 0.292	0.043	0.866
283	0.716 ± 0.419	0.247 ± 0.299	0.038	0.856
285	0.734 ± 0.415	0.251 ± 0.293	0.021	0.87
287	0.726 ± 0.375	0.264 ± 0.287	0.013	0.878
288	0.656 ± 0.377	0.234 ± 0.281	0.046	0.843
290	0.676 ± 0.356	0.225 ± 0.294	0.013	0.878
291	0.706 ± 0.377	0.254 ± 0.292	0.02	0.88
292	0.605 ± 0.313	0.224 ± 0.260	0.025	0.865
293	0.548 ± 0.286	0.220 ± 0.217	0.036	0.858
331	0.170 ± 0.067	0.081 ± 0.045	0.002	0.888
332	0.334 ± 0.194	0.116 ± 0.110	0.015	0.88
338	0.147 ± 0.062	0.076 ± 0.040	0.019	0.863
339	0.157 ± 0.061	0.081 ± 0.039	0.005	0.873
341	0.132 ± 0.060	0.068 ± 0.036	0.044	0.834
